# Reports of Societies

**Published:** 1875-07

**Authors:** 


### Reports of Societies.

### THE ILLINOIS STATE MEDICAL SOCIETY.

*Twenty-fifth Annual Meeting, May* 18, 1875.

The 25th annual meeting of the Illinois State Medical Society was held in the Conservatory of Music, at Jacksonville, Ill., commencing May 18th, a large number of delegates being in attendance. The meeting was called to order by Dr. David Prince, Chairman of the Committee of Arrangements, who welcomed the members to the city of Jacksonville.

Dr. J. H. Hollister, President of the Society, responded in a few appropriate remarks, after which the programme of exercises was announced by Dr. Prince.

On motion of Dr. T. D. Fitch, the Secretary, Dr. W. P. Pierce was chosen Treasurer *pro tem.* in the absence of Dr. Quine, the regular Treasurer.

On motion of Dr. Prince, the assessment was fixed at three dollars per member.

Dr. Prince also moved, that an initiation fee once paid should not be demanded again after a period of absence on the part of any member. After some discussion, the motion was carried. Subsequently, on Dr. Pierce asking for information as to whether, if a member had paid an initiation fee twenty years ago, and had been absent the intervening years, he was still to be considered a member, Dr. Fitch moved a reconsideration of the motion of Dr. Prince, which was carried—ayes 16, noes 10. On motion of Dr. Fitch, the order was so amended that a member loses his membership if he does not attend the meetings of the Society or pay his dues for a period of five years.

The Standing Committees were next called in their order, and the time fixed to hear such reports as were ready.

Dr. Whitmire, Chairman of the Committee on Nervous Diseases, announced that he had requested Dr. J. S. Jewell to prepare the report, and that he had prepared one.

The Secretary announced that Dr. Charles G. Smith, of Chicago, Chairman of the Committee on Dermatology, had not prepared a report, but desired to be continued on the committee another year. The request was granted.

A report of Dr. J. N. Niglas, of Peoria, on Hernia, was referred to the Committee on Surgery.

Dr. J. W. Freer announced a volunteer paper on the subject of Transfusion of the Blood.

Dr. Hoyt, of Bloomington, announced a paper prepared by Dr. E. W. Gray, on Small-pox, together with a series of resolutions on vaccination, which he asked the Society to adopt. It was referred to the Committee of Arrangements.

Dr. Rumbold, of St. Louis, announced a volunteer paper on "The Functions of the Uvula," which was ordered to be read at the convenience of the Society.

The Secretary announced that Prof. S. Curtiss, of Chicago, had prepared a paper on "The Anatomy of the Serous Membranes as affecting their Pathology." It was ordered to be read.

A communication was read from the Esculapian Medical Society, requesting that Dr. William Massey's essay on Insanity be read before the Illinois State Society. That gentleman was accordingly invited to read his paper, and this was the first paper read. It was followed by the reading of Dr. J. B. Walker's essay on Scarlatina, which was referred to the Committee on Publication.

The next thing was the reading of the paper of Dr. Curtiss on the Anatomy of the Serous Membranes. After some discussion of the subject, by Drs. J. W. Freer, Curtiss and Worrell, the paper was referred to the Committee on Publication.

On motion of Dr. Worrell, the paper of Dr. Massey was referred to the Committee on Publication.

In the afternoon, at 2 o'clock, the Association visited the Deaf and Dumb Asylum, and the Institution for Feeble Minded Children, spending the time there until 5 o'clock.

At 7½ o'clock in the evening the Society reassembled, and Dr. J. O. Hamilton, of Jerseyville, read a paper on the Diseases of Children, which was followed by one on the same subject, prepared by Dr. C. W. Earle, of Chicago, read by the Secretary.

After a pretty full discussion of the papers by Drs. E. F. Ingalls, Hamilton, Steele, Fitch, J. H. Mosher, Slater, Cowen, Worrell, Rumbold, and R. C. Hamill, the papers were referred to the Committee on Publication.

Dr. Rumbold, of St. Louis, read his paper on "The Functions of the Uvula," which was referred to the Committee on Publication.

Dr. David Prince next delivered an address on the use of electricity in therapeutics and surgery, accompanying the address by experiments with electrical machines. He was requested to prepare a paper on the subject for publication in the Transactions.

On motion of Dr. Freer, the proposition of Prof. Sanders, of the Conservatory of Music, to entertain the Society with a concert, was accepted, and the time fixed for Wednesday evening.

Adjourned.

---

WEDNESDAY, MAY 19.

The Society was called to order at 8 o'clock. Dr. J. W. Freer read his paper on Transfusion, which was referred to the Committee on Publication.

Dr. T. D. Fitch announced, that, in connection with the report of the Committee on Obstetrics, he would present some remarks on several new gynæcological instruments.

A recess of ten minutes was then taken, to allow of the selection of a Nominating Committee.

After the recess, Dr. E. B. Cooke submitted his report on Practical Medicine, which was followed by a paper by Dr. R. C. Hamill on the Treatment of Rheumatism by Phytolacca and Guiaca. The papers were discussed by Drs. Steele, Cooke, Birney, Herriott, Wright, Hay, and Edgar, of St. Louis, and were referred to the Committee on Publication.

Dr. E. Powell next read the report of the Committee on Surgery, which was followed by one on Hernia by Dr. E. L. Herriott.

Dr. David Prince exhibited an instrument for taking up the stitch in operations on soft palate and in skin planting.

Adjourned.

In the afternoon, the Society met at 2 o'clock, to witness the operation of transfusion performed upon a dog, by Dr. J. W. Freer, aided by Drs. E. Powell and Hay.

At the conclusion of this, the members took the street cars, and visited the State Hospital for the Insane, and the Asylum for the Blind.

In the evening, the President, Dr. J. H. Hollister, delivered his annual address before a large audience, the platform being occupied by the ex-Presidents present, Drs. Hamilton, McFarland, Worrell, Steele, Haller, and Prince. At the conclusion of the address, Dr. Prince offered a resolution, expressing the thanks of the citizens of Jacksonville for the pleasure they had in listening to the eloquent and scholarly address of the President. The resolution was unanimously adopted. The remainder of the evening was devoted to a concert given by the Illinois Conservatory of Music.

---

THURSDAY, MAY 20.

The Association reassembled at 8 A. M.

The paper of Dr. Niglas on Hernia, was referred to the Committee on Publication.

Dr. W. P. Pierce read a paper on Fractures, which was referred to the same committee.

Dr. Haller, chairman of the Nominating Committee, submitted his report, which, after some discussion and amendment by the committee, was presented as follows:

*Report of the Nominating Committee which was adopted.*

OFFICERS FOR THE ENSUING YEAR.

*President.*

THOMAS D. WASHBURN, M.D., Hillsboro.

*1st Vice-President.*

J. L. White, M.D., Bloomington.

*2nd Vice-President.*

John Wright, M.D., Clinton.

*Treasurer.*

John H. Hollister, M.D., Chicago.

*Permanent Secretary.*

T. Davis Fitch, M.D., Chicago.

*Assistant Secretary.*

C. B. Johnson, M.D., Tolono.

STANDING COMMITTEES.

*Board of Censors.*

E. Wenger, M.D., Gilman, *Chairman.*
E. Ingals, M.D.; Chicago.
E. S. Herriott, M.D., Grafton.

*Committee of Arrangements.*

S. H. Birney, M.D., Urbana, *Chairman.*
A. F. Darrah, M.D.
J. T. Pearman, M.D., Champaign.

*Practical Medicine.*

William Massey, M.D., Grandview, *Chairman.*
D. L. Jewett, M.D., Watseka.
I. W. Fink, M.D., Hillsboro.

*Committee on Surgery.*

Moses Gunn, M.D., Chicago, *Chairman.*
A. C. Rankin, Loda.
John G. Hays, Paris.

*Committee on Obstetrics.*

M. W. Walton, M.D., Ridott, *Chairman.*

C. Goodbrake, M.D., Clinton.

D. E. Foote, M.D., Belvidere.

*Drugs and Medicines.*

J. H. Etheridge, Chicago, *Chairman.*
T. J. Pitner, M.D., Jacksonville.
H. H. Hood, Litchfield.

*Necrology.*

G. W. Albin, M.D., Neoga.
William Chambers, M.D., Charleston.
J. N. Niglas, M.D., Peoria.

*Ophthalmology.*

E. L. Holmes, M.D., Chicago.

*Otology.*

F. C. Hotz, M.D., Chicago.

SPECIAL COMMITTEES.

*Diseases of Children.*

J. S. Williams, M.D., Otterville.

*Medical Jurisprudence.*

M. A. McClellan, M.D., Knoxville.

*Dermatology,*

J. Nevins Hyde, M.D., Chicago.

*Progress in Physiology.*

Sarah Hackett Stevenson, M.D., Chicago.

*Idiocy.*

C. T. Wilbur, M.D., Jacksonville.

*Neurology.*

Walter Hay, M.D., Chicago.

*Gynæcology.*

A. Reeves Jackson, M.D., Chicago.

*Hygiene.*

Jehu Little, M.D., Leroy.

*Diagnosis and Extirpation of Tumors.*

W. L. Goodell, M.D., Effingham.

*Empiricism.*

J. F. Todd, M.D., Galva.

*Prudence in the Administration of Therapeutic Agents.*

E. Ingals, M.D., Chicago.

*Uterine Displacements.*

T. D. Fitch, M.D., Chicago.

*Staphylorraphy.*

David Prince, M.D., Jacksonville.

*Electro-Therapeutics.*

Plym. S. Hayes, Chicago.

Place of meeting, Urbana, Champaign County.

The report was adopted.

Dr. T. D. Fitch submitted some new gynæcological instruments, with explanations. A discussion followed, which was participated in by Drs. Cooke, Edgar, of St. Louis, Frazier, and Boal.

Dr. David Prince also exhibited several similar instruments, with explanations.

Dr. Pierce read an abstract of a report on Surgery by Dr. Rood, of Lemont. The report was referred to the Publishing Committee.

Dr. E. Ingals, chairman of the committee, submitted his report on Necrology. It included memoirs of Drs. G. W. Young, of Aurora, and J. V. Z. Blaney, of Chicago.

The report on Nervous Diseases, by Dr. J. S. Jewell, was next received, and referred to the Publication Committee.

Dr. Prince declining to serve on the Committee on Galvano-Therapeutics for next year, his resignation was accepted, and Dr. William Hay, of Chicago, was appointed to prepare a paper on Electro-Therapeutics.

Resolutions were adopted requesting Congress to place the medical corps of the Army of the United States on an equal footing with the officers of other army corps.

Dr. T. D. Fitch read a report prepared by Dr. H. A. Johnson, of Chicago, on Phthisis, which was referred to the Committee on Publication.

The Secretary exhibited an improved stethoscope, which Dr. Curtiss explained.

The resolutions prepared by Dr. E. W. Gray, of Bloomington, on the regulation by a State enactment of the subject of vaccination in all public schools, asylums, etc., were read and adopted.

On motion of Dr. Haller, Dr. Prince was appointed a Special Committee on Staphyloraphy.

The Secretary submitted a report of the members present, and the Society adjourned until 2½ o'clock in the afternoon.

Society met at 2½ o'clock, as per adjournment.

The Treasurer's Report was read and accepted.

Also, the Secretary's Report, and the report of the Committee on Publication.

On motion of Dr. Freer, the Secretary was voted a salary of $100.

After some discussion in regard to the delay in printing the Transactions, Dr. Hay submitted the following, which was adopted :

*Resolved*, That at the next, and at all subsequent meetings, all reports of committees, and other papers designed for publication in the Transactions of this Society, be furnished in form ready for publication, and be placed at the disposal of the Publication Committee.

The following named persons were appointed delegates to the next session of the American Medical Association :

*Delegates to the American Medical Association.*

S. C. Plumm, M.D., Rock Island.
Walter Hay, M.D., Chicago.
J. F. Todd, M.D., Galva.
J. B. Rood, M.D., Lemont.
D. E. Foote, M.D., Belvidere.
F. B. Haller, M.D., Vandalia.
J. W. Freer, M.D., Chicago.
J. H. Hollister, M.D., "
Moses Gunn, M.D., "
W. P. Pierce, M.D., Lemont.
N. S. Reed, M.D., Chandlerville.
H. B. Buck, M.D., Springfield.
Thomas J. Whitten, M.D., Irving.
Wm L. Goodell, M.D., Effingham.
David Prince, M.D., Jacksonville.
G. Wheeler Jones, M.D., Danville.
J. Adams Allen, M.D., Chicago.
E. P. Cook, M.D., Mendota.
A. T. Darrah, M.D., Urbana.
M. W. Walton, M.D., Ridott.
M. Sheppard, M.D., Pason.
C. Goodbrake, M.D., Clinton.
J. O. Hamilton, M.D., Jerseyville.
Lester Curtis, M.D., Chicago.
T. D. Fitch, M.D., "
E. R. Willard, M.D., Wilmington.
W. A. Haskell, M.D., Edwardsville.
Robert Boal, M.D., Peoria.
C. T. Wilbur, M.D., Jacksonville.
J. M. Steele, M.D., Grandview.
L. Clark, M.D., Rockford.
O. Everett, M.D., Dixon.
T. D. Washburn, M.D., Hillsboro.
Wm. A. Elder, M.D., Bloomington.
C. Paul Simon, M.D., Chicago.
E. Ingals, M.D., "

*Iowa State Medical Society.*

Walter Hay, M.D., Chicago. T. D. Fitch, M.D., Chicago.

*Missouri State Medical Society.*

F. B. Haller, M.D., Vandalia.

*Indiana State Medical Society.*

W. W. McMann, M.D., Gardner.

*Minnesota State Medical Society.*

S. C. Plumm, M.D., Rock Island.

*New York State Medical Society.*

W. P. Pierce, M.D., Lemont.

*Nominating Committee.*

J. W. Freer, M.D., Cook County.
J. H. Mosin, M.D , Whiteside "
D. E. Foote, M.D., Boone "
T. N. Stewart, M D., Scott "
F. B. Haller, M.D., Fayette "
E. Wenger, M.D., Iroquois "
D. Lichty, M.D., Ogle "
L. B. Slater, M.D., Christian "
A. P. Tenney, M.D., McLean "
Fred. Cole, M.D., Woodford "
J. V. Frazin, M.D., Mercer "
J. N. Allen, M.D., Brown "
J. L. Hays, M D., Edgar "
D. H. Alvis, M.D., Henry "
J. S. Williams, M.D., Jersey "
W. H. Veatch, M.D., Greene "
D. L Wood, M.D , La Salle County.
M. W. Seaman, M.D., Macoupin "
W. H. Haskell, M.D., Madison "
M. W. Walton, M.D., Stephenson "
Sam'l Mileham, M.D., Adams "
P. H. Garretson, M.D., McDono' "
T. J. Maxwell, M.D., Henderson "
G. W. Albin, M.D., Cumberland "
W. L. Goodell, M.D., Effingham "
A. T. Darrah, M.D., Champaign "
W. G. Cochran, M.D , De Witt "
C. T. Wilbur, M.D., Morgan "
Geo. W. Wright, M.D., Fulton "
J. G. McKinney, M.D., Pike "
T. H. Whitten, M.D., Montgomery County.

A resolution expressive of the gratification of the members with their treatment by the citizens of Jacksonville, also, a resolution thanking the President, Dr. Hollister, for the skill and wisdom he had displayed as presiding officer, were unanimously adopted; and after a few farewell words from the President, the Society adjourned.

---

### CHICAGO SOCIETY OF PHYSICIANS AND SURGEONS.

*Regular Meeting, May* 10, 1875.

(Reported by E. Warren Sawyer, M.D.)

The Society met at the Grand Pacific Hotel. The President, Dr. Bartlett, in the chair.

This being the annual meeting, the yearly report of the Secretary and Treasurer was called for and read.

The Society then proceeded to the election of officers for the ensuing year, with the following result:

President, Thomas Bevan, M.D.; Vice-President, R. C. Hamill, M.D.; Secretary and Treasurer, Ralph E. Starkweather, M.D.; Recorder, E. Warren Sawyer, M.D.; Censors, Drs. Bartlett, Hyde, and Jackson.

Upon retiring, the President delivered the annual address, the publication of which was called for by the Society. It will appear shortly.

REPORT OF THE SECRETARY OF THE CHICAGO SOCIETY OF PHYSICIANS AND SURGEONS, FOR THE YEAR ENDING MAY 10, 1875.

*Mr. President and Gentlemen:—*

I have the honor to submit herewith the Annual Report of the Secretary of the Society for the third year since its organization.

On the 11th of May, 1874, there were fifty-nine members in active co-operation with the Society, and two non-residents, whose names were carried on the roll. Since that date three have removed from the city, and twenty-one have united with us. This leaves eighty-two names of members on the active list. One has been by vote declared an associate member. Deducting this name, with those who have ceased to reside here, from the entire number, we have seventy-eight names of physicians, in and about the city, who are actively interested in the work of the Society.

We have held twenty-three regular meetings during the year, and one special, making an average of two for each month, with a total attendance of 479, the average attendance upon each meeting being therefore nineteen.

The following reports have been made during the year just closed:

1. On the Absorption Bands of Blood in the Spectroscope.
2. On the Relative Mortality of Surgical Operations in the United States and other Countries.
3. On the Relation of Alcoholism and Sexual Diseases to Climate.
4. On the Ætiology and Pathology of Cholera Infantum.
5. On Asiatic Cholera: being a detailed account of its ingress and extension through portions of the United States during the summer of 1873.
6. On a New Method of Introducing Barnes' Dilators.
7. On the Action of Hydrate of Chloral upon the Blood.
8. On Pneumatic Aspiration: its methods and instruments.
9. On General Therapeutics.
10. On Carcinomatous and Scirrhous Neoplasms, (with microscopical illustrations).
11. On the Organic Alkaloids of the Cinchona Barks.

The following papers have been read before the Society during the year:

1. On the Microscopy of the Brains of the Insane, (with illustrative drawings).

2. On the Use of Podophyllin in Acute Rheumatism, (pub. Am. Jour. of Med. Sci.)

3. On the Eucalyptus Globulus.

4. On the Relative Value of Thermometers for medicinal purposes offered to the Profession.

5. On Puerperal Convulsions, (with illustrative case).

6. On the Management of the Third Stage of Labor.

7. On the Employment of Phytolocca Decordia and Guiacum in Rheumatic Diseases.

8. On the Action of Salycylic Acid.

The following translations of papers by foreign authors were also presented and read :

1. On the Therapeutic Value of Ipecac in Pulmonary Sweats.

2. On the Examination of the Mucous Membrane of the Uterus, (with illustrative microscopic drawings).

The following cases have been reported :

1. Case of Double Vagina and Uterus, with successful delivery of living child.

2. Case of Chronic Pyelitis.

3. Case of Subacute Meningitis.

4. Case of Amenorrhœa.

5. Case of Dysmenorrhœa, relieved by topical application of Faradic current.

6. Cases of Relapsing Fevers.

7. Cases of Obscure Neuroses.

8. Case of Retained Menses from Vaginal Constriction, successfully relieved by surgical interference.

9. Cases from Clinic for Diseases of Heart and Lungs—South Side Dispensary.

10. Cases of Subinvolution of Uterus, relieved by topical application of nitric acid.—In the "*Wom. Hosp.*, *State of Ill.*"

11. Case of Retention of Placenta during twelve days.

12. Case of Suppurative Pleuritis, relieved by aspiration.

13. Two Cases of Epilepsy successfully treated.

14. Case of Scirrhus of the Stomach, with details of post-mortem appearances and microscopic pathology, (illustrated by stained sections under the microscope).

15. Cases of Uterine Inertia, relieved by actæa racemosa.

16. Case of Aspiration of the Pericardial Sac—partial relief —death—and exhibition of specimen.

17. Two Cases of Fracture of Arm and Leg—(St. Luke's Hosp.)

18. Case of Multilocular Ovarian Cyst, successfully treated by electricity and acupuncture.

19. Fatal Case of Corroding Ulcer of the Uterus.

20. Case of Traumatic Epileptiform Convulsions.

21. Case of Variola, aborted by employment of sulphite of soda and carbolic acid.

22. Four Cases of Land Scurvy, successfully treated in Cook County Hospital.

23. Two Cases of Uterine Fibroid Tumors, relieved by hypodermic injection of ergotine.

24. Case of Cerebro-spinal Meningitis Simulating Hydrophobia.

25. Case of Successful Removal of Colloid Multilocular Ovarian Tumor, followed by death; with chart of pulse and temperature record, and exhibition of specimen.

26. History of a singular Case of Catalepsy, treated in Mercy Hospital.

The following specimens have been presented during the year —in addition to those already mentioned:

1. Nine calculi, removed from patient at a single operation.
2. Horn, removed from the forehead of a woman 55 years old.
3. Portable Electro-Faradic Machines, with chloride of silver battery.
4. Tumor of Dentine.
5. Cast of an anencephalous monster.
6. A novel inhaler for anæsthetic purposes.

During the year, Art. I of the Constitution has been amended so as to exclude candidates for membership who have not been in actual practice for two years, and who may be interested in the sale of medicines and surgical appliances. Two sections have also been added to Article IV, providing for the election of Associate and Honorary Members; while a new By-Law has been added, restricting the privilege of each member to the nomination of but one candidate during the year.

Two important resolutions have been passed, one recommending the delegates of the Society to both the State and National

Medical Associations to urge upon those bodies the memorializing Congress with reference to the remission of the duties on imported scientific works ; the other fixing the Annual Meeting as the appropriate date for the appointment or election of such delegates.

In accordance with a resolution of the Society, passed Dec. 28, 1874, a committee has been appointed to wait upon the President of the Society, and, in accordance with his expressed wish, examine his specimens of the fungus from the Mississippi river ague bottoms, and report in full to the Society upon the same, as well as upon the paper prepared and read by Dr. Bartlett, on the 10th of April, 1873. I am informed by the chairman of the committee, that they have waited upon Dr. Bartlett, and arranged upon a date for the investigation proposed.

The protracted work, and subsequent report of the committee on the relations between physicians and pharmicists are fresh in your recollection, and require no word of comment from one who co-operated with them in detail. It is sufficient to note that the result obtained was all that any reasonable individual could have anticipated at the outset of the inquiry. It awakened a sentiment in the minds of many men connected with the two professions, which led them to define for themselves a distinct boundary line between what should and what should not be permitted in the light of the highest ethics.

Nor is it necessary to review the part taken by this Society in the banquet extended by the Medical Profession of the city to the State Association, at the date of its last annual convention in our midst. The large representation of the Society in the number that enjoyed the festivities of that occasion, and the able address of our President, are remembered by many who are gathered here to-day. Were the recollection wanting, the details published in the Transactions of the Medical Society of the State of Illinois for 1874, would vividly recall the occasion. It is proper to add that that was the largest social gathering of the representatives of the Medical Profession of the city of Chicago that has ever occurred, and so great was the harmony and good feeling fostered by the amenities of the banquet, that not a few have felt it would be a desirable end accomplished if some such social reunion could occur annually.

The thanks of the Society during the year concluded are due to the editors of the two Medical Journals of Chicago for their kindness in publishing fully all the transactions and papers of the Society that have been submitted to them. The resolution passed Nov. 23, 1874, requiring that thereafter only the strictly scientific and literary transactions of the association should be spread before the public—reserving for the records, the executive and other acts—have improved the character of the published material.

In this connection it is proper to state, that for the accuracy and regularity with which all such matter has been prepared for publication, the Society itself, and the editors of the Medical Journals no less, are equally indebted to the faithfulness of the Reporter for the year, Dr. R. E. Starkweather. When it is considered that he has been under the necessity, during the entire year, of preparing duplicates of each report of the transactions —one for each Journal—and that these reports have frequently embodied long debates and abstracts of extended papers and verbal reports, an idea may be had of the actual labor involved in the discharge of his duties. How efficiently he has performed his task you have been in position to determine, who have examined the weekly and monthly exhibits of the medical press.

To Dr. F. H. Davis, the editor and publisher of the Medical Examiner, the Society is indebted for a bound copy of all the papers and transactions published in that Journal during the year 1874. These were collected by Dr. Davis himself, from the mass of material which was at his disposal, and bound in this convenient shape for the purpose of presenting them to the Society.

In concluding this resume of the work accomplished during the past year, it will be apparent, even upon a hasty glance over the field, that the character and amount of original investigation which has been encouraged in our midst, are sufficiently gratifying. It is also sufficient to suggest the conclusion, that he who has lost the opportunities afforded here for actual advance in the directions to which the reports have pointed, has failed to secure the absolute advantages provided by the Society. If some there be of this class, they have but one consolation left them—and that is, the ability to offset this loss by equally solid advantages gained elsewhere. The species of moralizing which is suggested by the arrival at such anniversaries as the present, is at once natural and necessary. The question comes home to each individual upon such occasions, "Have I, in a satisfactory manner, discharged my duties, not to the medical society, but to myself, during the period which has elapsed?"

Now just here comes in the operation of a principle, which is clearly admitted by all men and yet which they are quick to lose sight of. Perhaps, in consideration of the fact that, to-day after serving in the capacity of Secretary for three years, I resign my office to the hands of another, I may be pardoned for a representation of it.

It is a common enough error to conclude that the benefit to be derived from an association of this kind, is obtainable by attendance upon its meetings, listening to the papers read and the discussions which result, and digesting the accumulated information so as to make it the property of the hearer. It is undeniably true that benefit is to be gained by this process, but so inferior is it to the larger improvement of another kind placed within the reach of all, that the end would not actually justify the expenditure of the time and trouble necessary to secure it.

The most valuable of the advantages afforded by a medical society are certainly *not* those resulting from the information to be acquired at its meetings. For the periodicals and permanent literature which provide the student with the fullest details upon every question, are accessible to each in the office and study. What extempore speaker among us can furnish such instructive pabulum as Murchison, Broadabent, Biesiodecki or Nelaton! What essayist of our number can compare in brilliancy and depth with Hutchinson, Bennet, Bernutz or Virchow! Shall we then abandon our efforts and cease to unite ourselves in the bonds of medical organizations?

The answer given by the thoughtful man will be a decided negative, and his reason will be this: The greatest benefit derivable from these associations accrues to the speaker, the writer, the exhibitor—and *not* to the hearer. It is he, who, having observed or amassed facts, comes to others with his treasure, and toils in the effort—it is he, I say, who obtains the great benefit of the deed, not he merely who receives his share of the treasure.

It is not the *receiving* which makes us richer, it is the giving. He who brings me a fact or an idea, is richer than I. It is the gladiator in the arena, whose muscles are hardened, whose courage is heightened, whose brain becomes cool and clear—not the listless or interested spectator on the benches.

The American poet, Lowell, has illustrated the truth of this universal law in one of the most finished of his productions. In the "Legend of Sir Lawful," the knight goes forth in quest of the Holy Grail, but returns at the conclusion of a vain and weary pilgrimage, utterly disheartened. It is only when he is quite near his home and divides his scanty crust with a miserable beggar, that he is rewarded by discovering the sacred object of his search.

The knight of the helmet, spear and shield has long been succeeded by the man with the microscope, the thermometer and the scalpel. But the latter, no less than the former, will fail to attain the precious end of his researches if he does not divide by the way his crust of truth with his fellows.

That member of this Society who faithfully discharges his duties to his associates during the ensuing year, will assuredly be the wiser and the stronger for the effort.

JAMES NEVINS HYDE.

CHICAGO, May 10, 1875.

---

### CHICAGO SOCIETY OF PHYSICIANS AND SURGEONS.

*Regular Meeting, May 24, 1875.*

(Reported by E. WARREN SAWYER, M.D.)

Prof. Thomas Bevan, President, in the chair.

Prof. Freer reported a series of experiments made upon dogs, with a view to ascertaining how long blood may be kept and afterwards safely transfused into dogs.

*First Experiment.*—10 oz. of venous, defibrinated dog's blood, was transfused into another's veins, the blood having stood for seven hours upon ice, but warmed to 95° F. just before the operation. No bad symptoms resulted.

*Second Experiment.*—8 oz. of arterial blood, which had been exposed for seventeen hours to the temperature of the laboratory, about 65° F., was transfused, with no ill effects.

*Third Experiment.*—This dog was first bled from the carotid artery till he could bleed no longer, about 20 oz. being let. Fresh blood was transfused, and dog recovered rapidly.

*Fourth Experiment.*—First bled to insensibility; respiration had ceased, 32 oz. escaping; 10 oz. of blood, which had previously stood upon ice for forty-eight hours, was transfused. Dog was resuscitated, but died seventeen hours afterward. This was an unfavorable subject for the operation, as it was a dog which had been caught up from the street at the moment, and was greatly frightened.

*Fifth Experiment.*—This dog was first bled 6 oz.; then 7 oz. of blood, which had been on ice for the previous seventy-two hours, was transfused. Dog recovered.

In all instances the blood used was defibrinated immediately after being drawn. With one exception, the blood was brought to 95° F. at the moment of transfusion. In this exceptional instance the blood was accidentally passed into the dog's veins cold, without any bad effect.

Dr. Freer has long been of the opinion that blood was not as highly vitalized as other tissues; he regarded it rather as food in its most highly prepared shape, and ready for assimilation. By transfusion the body can be nourished *direct*, independently of the digestive organs. The indication for this operation will be the threatened inanition of the patient. More people die from inanition than we realize. In pulmonary consumption, for example, the patient rarely dies from asphyxia, and very rarely from hæmorrhage, but from the failure of the digestive organs to furnish assimilable food—in other words, from starvation.

In caries about large joints, the patient ultimately succumbs, because the digestive function has failed long before. By supplying the readily assimilated food, in the operation of transfusion, we give the digestive organs rest; and, in some instances, we may hope for an arrest in the morbid changes, even in so formidable a disease as pulmonary consumption.

In answer to Dr. Starkweather. McDonald's apparatus was used in transfusion.

Answer to Prof. Etheridge. No unsuccessful cases thus far have occurred.

Dr. Simons remarked, that in cases of immediate danger of starvation, small quantities of this blood food might be given, from time to time, just as one would inject hydrate of chloral in the veins, or any other drug.

The President regarded the thought as a most important one ; for, in some instances of malignant disease of the digestive tract, which seems alone too slight to cause death, the threatened starvation might be arrested almost indefinitely.

In answer to Dr. Hamill, Dr. Freer said, that if a person should digest no food at all, it will not be necessary to resort to transfusion daily, for the good effects are not so transitory.

Dr. Simons suggested that it would be an interesting fact to determine by experiment, viz., how long a dog could be kept alive by continuous transfusion, with absolutely no food taken into the stomach.

It was moved by Dr. Hyde, and carried, that Prof. Freer be requested to read a more extended paper on the subject, and exhibit apparatus, details of the operation, etc., at some future day.

Dr. Lester Curtis read a paper on the histological structure of the serous membranes, with reference to certain pathological processes which they may be the seat of. The subject was illustrated with diagrams. The writer had been struck by the fact that the effect of inflammation of the serous membrane was quite unlike the effect of that process when the mucous membrane was attacked. An explanation of this disparity was to be found in the structure of these membranes. The epithelial covering of the serous membranes can be separated into two layers, between which coagulable lymph was effused, thus converting the membrane into a true granulating surface, which is always followed by contraction. Unlike this, the epithelial covering of the mucous membrane is not thus separable into two layers.

Dr. Henrotin reported the case of a lady who had suffered a scald of the feet about two weeks before. Everything was progressing favorably, the healing nearly completed, when she was seized, while sitting in her chair, with palpitation of the heart and syncope, followed by death in twenty minutes. During life the lady had had frequent attacks of palpitation of the heart and syncope; once a blowing sound was heard on the aortic orifice.

Dr. Hyde reported a case of pityriasis versicolor in a lady, which he felt emboldened to speak of, because two well known physicians of this city had treated her with various drugs in large quantities for liver disease, and finally for kidney trouble. A single warm bath and the local application of sulphate of soda in solution was all that was required.

Dr. Wood mentioned that he had seen thirty cases of this disease in recruits. He had not found it intractable under the use of corrosive sublimate externally.

Dr. Simons spoke of a case in which the hand was passed into the rectum for the purpose of diagnosis. The case was obscure; that of a man who passed masses of fæces through the urethra.

---

[From the Medical News and Library.]

### MEDICAL NEWS.

### DOMESTIC INTELLIGENCE.

*American Medical Association.*—This Association held its twenty-sixth annual meeting at Louisville, Ky., on the 4th, 5th, 6th and 7th of May of this year. The attendance we learn was very large, and the proceedings entirely harmonious.

*May* 4. The Association was called to order by Dr. E. Richardson, chairman of the Committee of Arrangements, who welcomed the delegates in an eloquent address.

The President, Dr. Bowling, then delivered the annual address ; the subject was Medical Education.

*5th.* Dr. J. M. Toner, chairman of the Committee to memorialize Congress on the rank of the medical staff of the army, made a report, and offered the following resolutions, which were adopted :

*Resolved,* That this Association learns with regret that no action was taken by the last Congress upon its recommendation in behalf of the Medical Department of the United States Army, and that we respectfully renew our petition, that Congress will enact such a bill for the benefit of the medical department of the army as will secure to its officers that share of rank and promotion to which we consider they are entitled, and which should be at least fully equal to that enjoyed by any other staff corps, or by the medical corps of any army.

*Resolved,* That a committee of five be appointed to call the attention of Congress to this subject, and the petitions which were forwarded to the last Congress by the physicians of the United States.

After the transaction of some further miscellaneous business, Prof. Gross, of Philadelphia, read an elaborate paper on Venesection as a Lost Art.

Prof. Austin Flint, chairman of the Committee on Practical Medicine, next read a report on the Medical Discoveries during the Past Year, in which he discussed the subjects of alcoholism, motor centres, new remedial agents, transfusion, and the natural history of crime. The report was referred to the Publishing Committee.

Dr. Toner made a report, suggesting the organization of an international medical association, in which the profession of America should be represented at the meeting in Brussels. Referred to the Committee on Nominations.

*6th.* Delegates were appointed to repreesnt the Association in the International Medical Association, to be held in Brussels, in September next, and to confer with a committee from the Canadian Medical Association, which is to meet in Halifax, Aug. 5th next, on the subject of holding an international convention of the two associations.

The reports of the Committee of Publication, of the Treasurer, and Librarian, were severally read.

Dr. S. D. Seelye, of Alabama, offered a prize of one hundred dollars for the best essay on Bright's Disease, which was accepted.

Dr. J. Marion Sims, as chairman of the special committee appointed to devise plans for the establishment of the McDowell memorial fund, offered the following preamble and resolutions, which were adopted :

WHEREAS, It is universally acknowledged that the late Ephraim McDowell, of Kentucky, was the originator of ovariotomy ; and

WHEREAS, We believe that proper measures should be instituted to commemorate this great achievement, and do appropriate honor to its author ; therefore

*Resolved*, That this Association recommend to each of its members, and to the profession generally, to contribute annually such sums as they may think proper until the amount of ten thousand dollars shall be accumulated, which shall be known as the McDowell Memorial Fund, the interest of which shall be devoted to the payment of prizes for the best essays relating to the diseases of the ovaries.

*Resolved*, That this fund shall be invested by trustees, to be appointed by the Association, and subject to such regulations as it may desire.

*Resolved*, That this Association shall elect a board of three trustees, whose duty it shall be to carry out the object of these resolutions, and whose term of office shall continue five years.

*Resolved*, That this Association will leave to the State of Kentucky the grateful privilege of providing a local memorial to the memory of Dr. McDowell.

Prof. Gross made some remarks regarding the justice of Dr. McDowell's claim to be the originator of ovariotomy, and announced that he would subscribe one hundred dollars to the fund.

Prof. Gross announced that it was designed to hold an international medical conference in Philadelphia during the Centennial celebration.

A report was received from the Judicial Council on various matters of ethics, after which Prof. E. M. Moore, of Rochester, N. Y., read an elaborate paper on Transfusion.

Prof. Byford then read a paper on Uterine Fibroids, which was referred to the Publishing Committee.

*7th.* After the transaction of some miscellaneous business, including the appointment of certain committees, the Nominating Committee reported the following officers for the next year, which nominations were confirmed :

*President*—J. Marion Sims, M.D., of New York.

*Vice-Presidents*—First, John D. Jackson, M.D., of Kentucky; second, Samuel Lilly, M.D., of New Jersey; third, N. Pinckney, M.D., of U. S. Navy; fourth, S. D. Seelye, M.D., of Alabama.

*Treasurer*—Caspar Wister, M.D., of Pennsylvania.

*Librarian*—Wm. Lee, M.D., of District of Columbia.

*Committee on Library*—Johnson Eliot, M.D., of District of Columbia.

*Assistant Secretary*—Richard J. Dunglison, M.D., of Pennsylvania.

*Committee of Arrangements*—Drs. Wm. Pepper, chairman; Frank Maury, Albert Fricke, A. Hewson, S. W. Gross, William Goodell, and T. M. Drysdale.

*Committee on Publication*—Drs. F. G. Smith, T. M. Drysdale, Albert Fricke, and William B. Atkinson, of Philadelphia.

*Officers of Sections*—Practice of Medicine, Materia Medica, and Physiology, F. G. Smith, Pennsylvania, chairman; B. A. Vaughn, of Mississippi, secretary. Obstetrics and Diseases of Women, Samuel C. Busey, of District of Columbia, chairman; R. Battey, of Georgia, secretary. Surgery and Anatomy, Alonzo Garcelon, of Maine, chairman; E. T. Easley, of Texas, secretary. Medical Jurisprudence, Chemistry, and Physiology, E. L. Howard, of Maryland, chairman; E. L. Hurlburt, of Illinois, secretary. State Medicine and Public Hygiene, B. C. Kedzie, of Michigan, chairman; Ezra M. Hunt, of New Jersey, secretary.

*Judicial Council*—The terms of a portion of the Judicial Council expiring at this meeting, the following were appointed to take their places: Levin S. Joynes, Virginia; R. N. Todd, Indiana; Robert Battey, Georgia; James E. Morgan, District of Columbia; T. B. Flager, New Jersey; S. N. Bentram, Pennsylvania; A. Dunlap, Ohio.

The following were appointed a committee on the Prize Essays: Drs. Samuel D. Gross, F. G. Smith, Alfred Stille, E. Wallace, H. C. Wood, Pennsylvania.

Dr. Bowditch, of Boston, made some extended remarks upon the practicability of establishing a National Council of Health, and offered the following resolution:

*Resolved*, That each year, until otherwise ordered, the President elect and the Permanent Secretary be directed to appeal, in the name of this Association, to the authorities of each State, where no State Board of Health exists, urging them to establish such boards, and that the Permanent Secretary be directed to report the names of the States where Boards of Health exist, and also those which decline to establish them.

After several complimentary speeches were delivered, and the adoption of resolutions of thanks for hospitalities received, the Association adjourned to meet in Philadelphia on the first Tuesday of June, 1876.

---

*Association of American Medical Editors.*—This Association held its annual meeting at Louisville, May 3rd. The President, Dr. Edgar, delivered the annual address. The subject of it was Medical Advertising and Medical Education.

The following officers were elected for the ensuing year: President, Dr. Bell; Vice-President, Dr. H. C. Wood; Secretary, Dr. F. C. Davis.

